# Incidence of malignancy in patients with common variable immunodeficiency according to therapeutic delay: an Italian retrospective, monocentric cohort study

**DOI:** 10.1186/s13223-020-00451-z

**Published:** 2020-06-26

**Authors:** Veronica Pedini, Jacopo Umberto Verga, Irene Terrenato, Denise Menghini, Cristina Mezzanotte, Maria Giovanna Danieli

**Affiliations:** 1 Medical Clinic, United Hospitals and DISCLIMO, Polytechnic University of Marche, Ancona, Italy; 2 Medicine Departement, Destra Secchia Hospital, Pieve di Coriano, ASST Mantova, Mantua, Italy; 3 Molecular and Applied Biology, Polytechnic University of Marche, Ancona, Italy; 4 Biostatistic and Bioinformatic Unit, Scientific Direction, IRCCS Regina Elena National Cancer Institute, Rome, Italy

**Keywords:** Common variable immunodeficiency, Diagnostic delay, Therapeutic delay, Ig replacement therapy

## Abstract

**Background:**

Common variable immunodeficiency (CVID) is the most common symptomatic primary immunodeficiency and has a broad spectrum of clinical manifestations. Among non-infectious complications, an increased incidence of malignancies may have a special relevance for survival, but little is known about treatment efficacy on malignant complications.

**Methods:**

This was a monocenter retrospective study on CVID patients, designed to provide preliminary data for the investigation of the possible link between therapeutic delay and tumor incidence.

**Results:**

A total of 67 CVID subjects were included. The median diagnostic delay was 7.5 years (range: 0–63 years), and the median therapeutic delay was 8.5 years (range: 0–67 years). Malignancies were diagnosed in 18 (27%) patients. Eight out of 18 (44%) patients with a malignancy had lymphoma. Patients who developed a malignancy showed a longer therapeutic delay in comparison to patients with no malignancy, although no statistical significance was achieved (11 years vs 8 years, respectively, p = 0.424). We observed a lower frequency of malignancy in CVID patients with reduced therapeutic delay compared with patients with therapeutic delay ≥ 10 years. With a therapeutic delay of > 1 year, 74% had no tumor, and 25% had a tumor; with a therapeutic delay of > 10 years, 65% had no tumor and 35% had a malignancy. Among patients who had no malignancy, 64% had a therapeutic delay of < 10 years, and 36% had a therapeutic delay of ≥ 10 years. Among patients with malignancy, 47% of subjects had a therapeutic delay < 10 years, and 53% a therapeutic delay ≥ 10 years.

**Conclusions:**

The observation of clinical characteristics of our patients with CVID may suggest that an early institution of IgG replacement therapy could be of benefit for the prevention of malignant complications.

Name of the registry: Comitato Etico Regionale delle Marche. Trial registration number: 1505. Date of registration: 27/10/2016, Retrospectively registered URL of trial registry record: http://www.ospedaliriuniti.marche.it/portale/archivio13_cerm-ancona_0_446_1.html. The trial was not registered before the first participant was enrolled

## Introduction

Common variable immunodeficiency (CVID) is the most common symptomatic primary immunodeficiency in adults and is characterized by reduced serum levels of immunoglobulin (Ig) G, IgA and/or IgM. Specific antibody production is reduced, or absent and several immune cell defects have been recognized in CVID patients [1, 2].

CVID has a broad spectrum of clinical manifestations, which may be gathered into two main phenotypes, possibly needing differential management: one group of patients are predominantly affected by recurrent infections and a second group of patients, who have a poorer prognosis, have autoimmune/inflammatory manifestations [3–5].

Among non-infectious complications, an increased incidence of malignancies may have a special relevance for survival. A higher frequency of malignancy in CVID patients compared with the general population, with a 5–12-fold risk increase, has been reported [6, 7]. In a recent meta-analysis, the prevalence of malignancy in CVID patients was found to be 8.6%, with a prevalence of lymphoma of 4.1% [8]. The clinical spectrum of non-infectious complications has been recently described in a cohort of 623 patients with CVID in the USA [2]. In this cohort, lymphomas had an incidence of 6.7%, and other tumors had an incidence of 6.4%. Lymphomas (all were B cell type except one subject) were diagnosed more often in females than in males (p = 0.036) [2]. Remarkably, as reported by Odnoletkova et al., the prevalence of solid tumors in 2700 European patients with CVID significantly correlates with diagnostic delay (HR: 1.08, 95% CI 1.04–1.11; p < 0.0001) [9]. In particular, each year of diagnostic delay was associated with an increase of the risk of solid tumors by 8%.

While IgG replacement therapy has effectively improved the prognosis of CVID patients with prevalent infectious diseases, to our knowledge no study has tried to correlate delayed institution of replacement therapy (hence, therapeutic delay) with the onset of neoplasms [2, 10]. To address this issue, the relationship between therapeutic delay and cancer incidence should be studied. The clinical pattern, risk factors and the pathophysiological mechanisms of malignancies in subjects with CVID need to be thoroughly described, to prompt investigation on the possible preventive role of therapy.

This monocenter retrospective study on CVID patients, was designed to provide preliminary data for the investigation of the possible link between therapeutic delay and tumor incidence.

## Patients and methods

This was a monocenter retrospective study, carried out at Medical Clinic, Ancona, a referral university hospital in the centre of Italy. All patients’ data were recorded and analysed from our dedicated database. All patients previously gave informed consent to data collection and publication (Prot. N. 20160561 OR, 27/10/2016; n. 871 DG, 7/12/2016). This specific study was further submitted for the approval of the local Ethical Committee (n. 1505).

Patients with CVID, aged ≥ 18 years, and followed-up for ≥ 1 year were included in the study. Patients were diagnosed with CVID according to the revised European Society for Immunodeficiency (ESID) criteria, and/or according to the International Consensus Document (ICON) criteria, for cases preceding ESID 2019 revision [1, 11]. They were treated and followed-up according to the current clinical practice relative to the time of their management. The onset of CVID was defined by the presence of the first finding related to the disease.

Clinical phenotypes were defined according to Chapel et al. [12]. The diagnosis of GLILD was based on clinical features, ventilatory restrictive pattern at lung spirometry, high-resolution computed tomographic imaging of the chest [13] and exclusion of other conditions. Even if our patients did not perform lung biopsy, this is not strictly necessary in specific clinical conditions [14]. Moreover, our patients underwent lymph node, cutaneous and liver biopsies documenting the presence of non-caseating granulomatous lesions. Enteropathy was detected by colonoscopy and subsequent histological examination.

### Assessments, follow-up and treatment

According to our practice, at first examination, we collected a full anamnesis for each patient, comprising complete past medical history, physiological anamnesis, familiarity for immunodeficiencies and autoimmune diseases and current medical situation, consanguinity among parents and grandparents. We also collected the interval among first symptoms and diagnosis (diagnostic delay) and start of treatment (therapeutic delay). Blood tests at the first visit included both routine ones and more specific examinations aimed at confirming the diagnosis of CVID, excluding a secondary immunodeficiency and predicting possible complications.

Clinical follow-up was performed every 3–6 months. At each control, all patients underwent a complete physical examination, including superficial lymph nodes and spleen evaluation. Patients were tested for Ig levels and routine analysis every 3–6 months and every 12–24 months for exclusion of emerging causes of secondary immunodeficiency.

In order to assess organ involvement, we performed at diagnosis: high-resolution chest CT scan and pulmonary function tests with CO diffusion capacity; esophageal gastroscopy with biopsy; abdominal ultrasound; oncological screening as for general population; other examinations (colonoscopy, thyroid ultrasound, etc.) in selected cases. These analyses were performed at follow-up as needed. All patients repeated at least annually pulmonary function tests, abdominal ultrasound and oncological screening.

As concerns Ig replacement therapy, patients received intravenous immunoglobulin (IVIg) or 20% subcutaneous immunoglobulin (SCIg, Hizentra ^®^ CSL Behring, King of Prussia, USA; starting from 2008, in the first years the 16% formulation was adopted) or facilitated-SCIg (fSCIg, Hyqvia ^®^, Takeda, Japan, starting from 2014) at the monthly dose of 0.4–0.6 g/kg. Side effects were registered during treatment in a diary provided to patients. Dose adjustments were based on infective recurrences and serum IgG levels. Goals of treatment were as follows: ≤ 2 infectious episodes/years and serum IgG levels ≥ 700 mg/dL. In case of recurrent infections despite appropriate serum IgG levels, antibiotic prophylaxis with macrolides was adopted (azithromycin 250 mg/day for 3 days weekly).

The aim of Ig replacement therapy is that to prevent infectious episodes since there is not a universally accepted serum IgG trough level [15, 16]. In our clinical practice, the target is a serum IgG levels around 700–800 mg/dL with differences between the Centers even within the same country [17, 18].

### Objectives

The primary objective was to evaluate the frequency of therapeutic delay, expressed as years in CVID patients with or without malignant complications. Information on diagnostic delay was also collected.

Diagnostic delay was defined as the time between the occurrence of the first CVID symptom (reported in clinical charts or estimated on patient’s memories) and the effective CVID diagnosis; and therapeutic delay as the time between the occurrence of the first symptom (reported in clinical charts or estimated on patient’s memories) and the initiation of IgG replacement therapy. Data were censored on 31 January 2020.

### Statistical analysis

Descriptive statistics were calculated for all variables of interest. Categorical data were presented as frequencies and percentage values, continuous variables as median values and their relative range. The Mann–Whitney nonparametric test and the Pearson’s Chi square test, when appropriate, were applied to test potential differences between groups. p < 0.05 was considered statistically significant. All analyses were carried out with SPSS (SPSS version 21.0, IBM, Armonk, NY, USA).

## Results

### Subjects and follow-up

A total of 67 subjects were included in the study, with a CVID diagnosis received between 1983 and 2019. Patients were followed-up at this center since January 2008 with a median follow-up of 50 months (range: 0–183 months). Main patients’ demographic and clinical characteristics at diagnosis are reported in Table 1. Median age at first CVID symptom occurrence was 30 years (range: 10–75 years), while median age at CVID diagnosis was 50 years (range: 16–79 years). At the onset, CVID patients presented with sinopulmonary tract infections (isolated, n = 58; associated with other features, n = 5), autoimmune cytopenia (n = 5) enteropathy (n = 2) and neoplasia (n = 2). GLILD was diagnosed in six patients. Lymph node (n = 3), cutaneous (n = 1) and liver (n = 2) biopsies were performed to document the presence of non-caseating granulomatous lesions. As for enteropathy, 11 patients presented collagenous colitis (n = 2) Crohn-like disease (n = 2) and colitis (n = 7).

**Table 1:** Characteristics of the patients at baseline

Criteria	n (%)*
No of patients	67 (100)
Gender	
Male	21 (31)
Female	46 (69)
Age at disease onset (years); median (min–max)	30 (10–75)
Age at diagnosis (years); median (min–max)	50 (16–79)
Diagnostic delay (years); median (min–max)	7.5 (0–63)
Therapeutic delay (years); median (min–max)	8.5 (0–67)

*Variables are represented by frequencies and percentage values, unless otherwise specified

A median diagnostic delay of 7.5 years (range: 0–63 years) was reported. At the time of data analysis, seven patients had died. Median serum Ig levels at diagnosis were IgG: 347 mg/dL; IgA: 31 mg/dL; and IgM: 28 mg/dL.

During our follow-up, several non-infectious complications occurred (Table 2). Organ-specific and systemic autoimmune manifestations were present in 30 (44%) patients. In all series, cytopenia was detected in 20 (30%) patients, among which 12 had immune thrombocytopenic purpura (ITP), and three had autoimmune hemolytic anemia. Granulomatous disease in any site occurred in 12 (18%) patients, among which six had granulomatous and lymphocytic interstitial lung disease, and eight had more than one organ involvement (lymph nodes, liver, skin). A CVID-associated enteropathy was found in 11 (16%) subjects, while *Helicobacter pylori* infection was demonstrated in 18 (27%) patients.

**Table 2:** Clinical features and kind of treatment at last follow-up visit in 67 common variable immunodeficiency patients

Criteria	n (%)
Non complicated patients	21 (31)
Cytopenia	
Autoimmune hemolytic anemia	3 (4)
Neutropenia	3 (4)
Immune thrombocytopenia	12 (18)
Autoimmunity	
Yes	30 (44)
Granulomatosis	
Yes	12 (18)
Granulomatous and lymphocytic interstitial lung disease	6 (9)
Granulomatosis in only 1 site	3 (4)
Granulomatosis in > 1 site	9 (13)
CVID-associated enteropathy	
Yes	11 (16)
*Helicobacter pylori* positivity	18 (26)
Allergies	
Overall	15 (22)
Drugs	12 (18)
Splenectomy	6 (9)
Therapy	
20% SCIg replacement therapy	28 (42)
Facilitated SCIg replacement therapy	17 (25)
IVIg replacement therapy	26 (38)

CVID: Common variable immunodeficiency; IVIg: intravenous immunoglobulin; SCIg: subcutaneous immunoglobulin

### Therapeutic delay

The median therapeutic delay was 8.5 (range: 0–67 years). A total of 64/67 patients received a therapy for CVID; subcutaneous IgG replacement was administered to 28 patients, facilitated subcutaneous Ig therapy was used in 17 cases, and intravenous administration was used in 26 patients. Due to previous reaction to IVIg, three patients received antibiotic prophylaxis with azithromycin.

### Malignancies

An oncologic disease was diagnosed in 18 (27%) patients, two of which had two distinct primary lesions (Table 3).

**Table 3:** Characteristics of malignancies in common variable immunodeficiency patients

Criteria	n (%)*
Cancer	
No	49 (73)
Yes	18 (27)
Lymphoma	8 (44)
Solid tumor	10 (56)
Age at cancer diagnosis (1) (years); median (min–max)	53 (21–85)
Age at cancer diagnosis (2) (years); median (min–max)	76 (41–85)
Time from first cancer diagnosis to CVID (months); median (min–max)	159 (10–692)

CVID: Common variable immunodeficiency

*Variables are represented by frequencies and percentage values, unless otherwise specified

Comparing patients who had malignant complications either before or during follow-up and those without tumors, the median age at CVID onset (29 years, range: 10–75 vs 30 years, range: 10–75), the median age at CVID diagnosis (52 years, range: 20–79 vs 46 years, range: 16–78), and Ig levels at CVID diagnosis (316 mg/dL vs 388 mg/dL) were similar, at the time of data analysis (Table 4).

**Table 4:** Comparison between cancer/no cancer patients and demographic/clinical characteristics

Criteria	Cancer		p-value
	Yes (n = 18)	No (n = 49)	
Age at CVID onset; median (min–max)	29 (10–75)	30 (10–75)	0.769
Age at CVID diagnosis; median (min–max)	52 (20–79)	46 (16–78)	0.449
Autoimmunity: Yes (n)	11	19	0.103
ITP: Yes (n)	5	7	0.202
Granulomatosis: Yes (n)	3	9	0.872
CVID-associated enteropathy: Yes (n)	2	9	0.477

CVID: Common variable immunodeficiency; ITP: Immune thrombocytopenic purpura; CVID onset was defined as the occurrence of the first findings related to CVID (see “Patients and methods” for details)

Overall, eight malignancies were diagnosed prior to CVID (mean time before diagnosis: 54 months), while the others occurred during the follow-up (mean time after CVID diagnosis: 160 months).

At diagnosis of malignancy (data available for 12 patients), the median level of IgG was 792 mg/dL (range: 506–1350); one patient was not receiving replacement therapy due to a previous severe adverse reaction. A malignant complication was the death cause for four out of seven dead patients.

Median age at the time of the first malignancy diagnosis was 53 (range: 21–85) years; while the median age at the time of a second malignancy diagnosis was 76 (range: 41–85) years (Table 3). Patients diagnosed with a malignancy before CVID, compared with those with tumors occurred after the CVID diagnosis, had a lower mean age at tumor diagnosis (43 vs 61 years, respectively), and a longer diagnostic delay for CVID (130 vs 49 months, respectively). Hypogammaglobulinemia had been found before the tumor occurrence in all eight patients with a malignancy preceding CVID, but not further investigated.

A total of eight out of 18 (44%) patients with an oncologic disease had a lymphoma (overall incidence: 12%): one mycosis fungoides, one large granular lymphocytic leukemia and six B-cell lymphomas (two were gastrointestinal lymphomas). No patient had both a lymphoproliferative malignancy and a solid tumor. Four lymphoproliferative malignancies occurred prior to the CVID diagnosis, and four during the follow-up, 4, 6, 7 and 21 years after the CVID diagnosis, respectively.

Ten patients had solid tumors and of these, two developed a second solid tumor. The 12 solid tumor types were: three melanoma, two thyroid cancers, one gastric schwannoma, two breast cancer, one pancreas cancer, two gastric cancers and one bladder cancer.

While the overall incidence of autoimmune disease in our case series was 44%, the condition was not equally distributed in patients with and without malignancy; in the absence of oncological complications the incidence was 38% (19/49 patients), but in the presence of malignancy it was 61% (11/18 patients). Most of the autoimmune diseases were represented by ITP, with an incidence of 14% and 27% (7/49 patients without malignancy, and 5/18 patients with malignancy).

### Malignancy and therapeutic delay

**Fig. 1:**
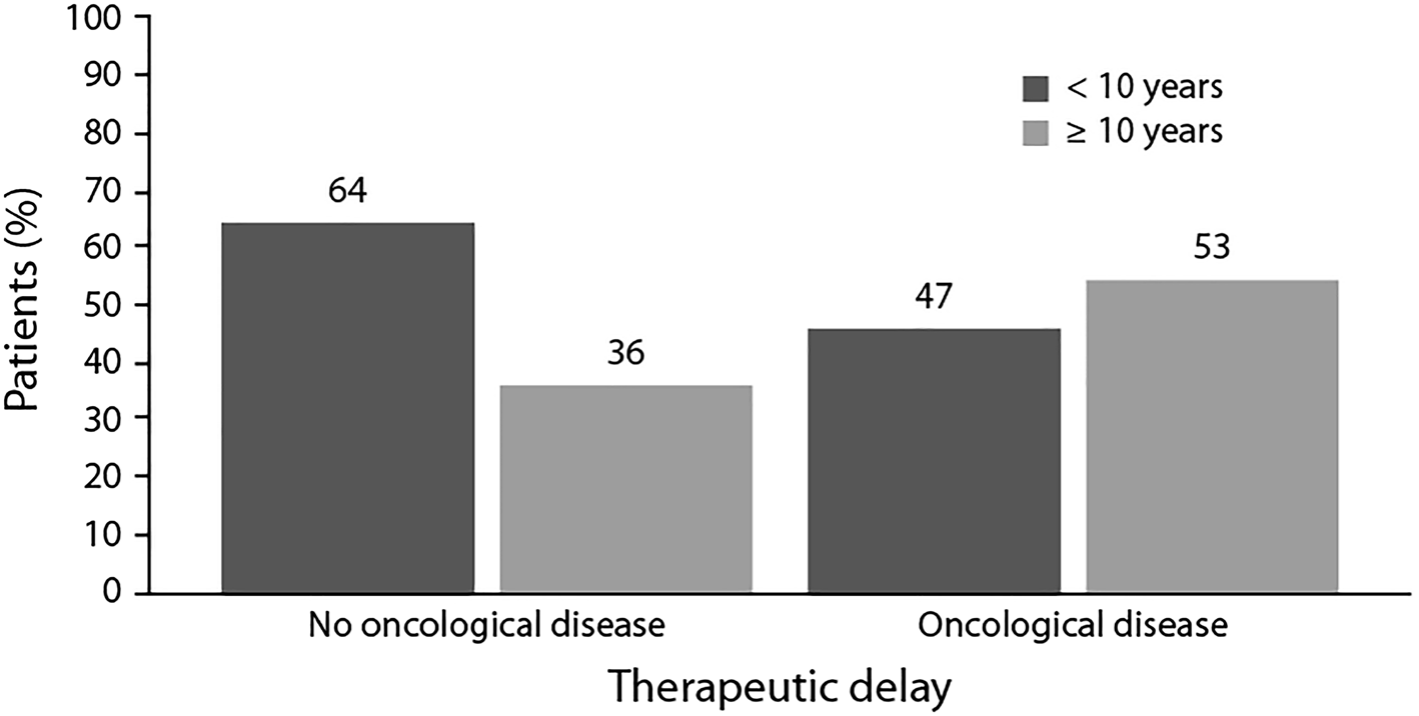
Incidence of therapeutic delays either ≤ 10 years or > 10 years in patients with malignancy or without malignant complications of CVID

The median therapeutic delay was 11 years in patients with malignancy vs 8 years in those without malignant complication (p = 0.424). Although a statistical significance was not achieved these results suggest a possible link between the therapeutic delay and tumor incidence (Table 5). Furthermore, among patients who had no malignancy at end of follow-up, 64% had a therapeutic delay of < 10 years, and 36% had a therapeutic delay of ≥ 10 years. Conversely, among patients with one or more malignancies, a therapeutic delay of < 10 years was recorded only for 47% of subjects, and a therapeutic delay of ≥ 10 years for 53% of subjects (Fig. 1a).

**Table 5:** Comparison between cancer/no cancer patients and therapeutic and diagnostic delays

	Cancer		p-value
	Yes (n = 18)	No (n = 49)	
Therapeutic delay (years); median (min–max)*	11 (0–67)	8 (0–50)	0.424
Diagnostic delay (years); median (min–max)*	11 (0–63)	5 (0–9)	0.579

*Times were calculated from the detected or estimated disease onset data

Similar observations were made for the diagnostic delay (Table 5 and Fig. 1b). The median diagnostic delay was longer in patients with malignancy compared to those who had no oncologic complication (11 years vs 5 years, respectively, p = 0.579). Among patients without malignant complications a diagnostic delay ≤ 10 years was reported in 69% of patients, while a diagnostic delay > 10 years was observed in 31% of patients without tumor; differently, patients with malignant complications had the same frequency of diagnostic delay ≤ 10 years and > 10 years. Among the 47 patients with a diagnostic delay of < 10 years, 74% had no malignancy, and 25% had it; among the 24 patients with diagnostic delay > 10 years, 62% had not an oncologic disease and 37% had it.

A comparison between cancer/noncancer patients and diagnostic and therapeutic delays categorized by different cut-off (sensitivity analysis) is reported as Additional file 1.

## Discussion

Mechanisms involved in the high susceptibility to malignancy of CVID patients are not totally clarified. Several mechanisms have been hypothesized to be involved, like genetic predisposition and genetic instability; persistent activation, and proliferation of the lymphoid cells during infections; decreased clearance of oncogenic viruses and bacterial infections.

In patients with CVID, the impaired immune response to infections is responsible for the chronic antigen stimulation, the chronic inflammation and the increased survival and proliferation of premalignant and malignant cells. It is, thus, interesting hypothesize that Ig replacement therapy with the consequent reduction of infections and related complications may decrease the risk of malignancy linked to the impaired immune response.

We have already described the potential anti-tumor activity of immunoglobulin, thanks to its immunomodulatory role due to its action in the adaptive immunity and in the maintenance of immune homeostasis (activation of FCGR; stimulation and NK cells cytotoxic activity on tumor cells; increased expression of pro-apoptotic molecules; reduction of tumor spread by Anti-VEGF Ab and reduced expression of metalloproteinases and anti-RGD Ab, etc.) [19]. However, to our knowledge, the correlation between delayed institution of Ig replacement therapy and the onset of cancer disease has never been investigated to date.

This retrospective observational study describes the clinical features of malignancies in 67 patients with CVID, followed-up for over 10 years in a single specialized center in Italy. The information obtained from this cohort of patients should be useful for further investigation on the effect of IgG replacement treatment on incidence and progression of tumors in CVID patients. We acknowledge that this study has several limitations, including its retrospective design, and the limited number of patients—however, in line with the rarity of the disease—which precluded us the possibility of a more refined statistical analysis taking into account all the variables. In addition, this was not an interventional study and can only suggest the relevance of the diagnostic and therapeutic delay. Last, in a limited number of cases (n = 5), information on therapeutic delay was collected based on patients’ memories We however believe that a careful description of a homogeneously followed-up population may nevertheless be interesting for researchers.

As expected, the incidence of malignancy and the proportion of lymphomas were high in our group of patients compared to the general population [6, 7]. In our group, 27% of patients had a malignant disease, with an incidence of lymphoma of 12%; so, we found higher incidences than those found by the meta-analysis by Kiaee et al. [8], but in line with other reports [20, 21].

Lymphomas were the most frequent malignancy, representing 40.5% of cancers in the meta-analysis by Kiaee et al., and 53.4% in the study by Resnick et al. [5, 8]. Our case series agreed with these data, as lymphomas were 44% of malignancies. Tumors were a very relevant cause of death in our cohort (four out of seven deaths), more than infections (two cases).

In our series, we documented a higher incidence of autoimmune cytopenia, in particular ITP, in patients who developed neoplasia than in those without malignant complications. It is possible that we observed very selected cases, with serious manifestations, as we operated in a tertiary care Centre. However, this data agrees with findings by Kralickova et al., who identified ITP as a potential risk factor for cancer development in CVID patients [22]. In addition, the observation further suggests that the immune dysregulation underlying CVID may promote malignant occurrence [22].

The main result of our study is that the median diagnostic delay was longer in patients with malignancy compared to those who had no oncologic complication and similar data were observed for the median therapeutic delay; although a statistical significance was not achieved probably due to the intrinsic limit of the study, that is limited sample size as a consequence of a rare condition, these results suggest a possible link between the diagnostic and therapeutic delay and tumor incidence. Furthermore, if these data were confirmed by a larger study, the information would support the hypothesis that the IgG replacement therapy could have a role in cancer prevention in CVID patients. Almost all the patients in this series received IgG replacement therapy, mainly through the subcutaneous route, but time of initiation was greatly variable, and a long period without treatment seems to impact on cancer risk.

Similarly, the diagnostic delay for CVID was found to be longer in patients with tumor onset previous to CVID diagnosis compared with those who had tumors after the CVID diagnosis. It is possible that malignancy was an early manifestation of undiagnosed CVID in these former subjects. Patients affected by the CVID phenotype characterized by a low incidence of infections and a prevalent incidence of non-infectious manifestations are at an increased risk of misdiagnosis, as clinician attention is not easily raised toward immune impairment in the absence of recurrent infection. In our case series, all patients with a tumor occurred before CVID diagnosis had been found with hypogammaglobulinemia even before tumor occurrence, but this clue had not been interpreted as an early symptom of CVID and it was not further investigated.

In conclusion, although with all the limitations of any retrospective study, the observation of clinical characteristics of our patients with CVID may suggest that an early institution of IgG replacement therapy could be of benefit for the prevention of malignant complications.

## Supplementary information

**Additional file 1: Table S1.** Comparison between cancer/no cancer patients and diagnostic and therapeutic delays categorized by different cut-off.

## Data Availability

The datasets used and/or analysed during the current study are available from the corresponding author on reasonable request.

## Abbreviations

CVID: Common variable immunodeficiency
Ig: Immunoglobulin
ESID: European Society for Immunodeficiency
ICON: International Consensus Document
ITP: Immune thrombocytopenic purpura
