# Supplementary material for: Incidence of malignancy in patients with common variable immunodeficiency according to therapeutic delay: an Italian retrospective, monocentric cohort study
Source: Allergy Asthma Clin Immunol. 2020 Jun 26;16:54. doi: 10.1186/s13223-020-00451-z (PMC7491341; doi:10.1186/s13223-020-00451-z)
Supplement: Supplementary file 1 — Additional file 1: Table S1. Comparison between cancer/no cancer patients and diagnostic and therapeutic delays categorized by different cut-off. [file 13223_2020_451_MOESM1_ESM.docx]

**Supplementary Material**

Table S1. Comparison between cancer/no cancer patients and diagnostic and therapeutic delays categorized by different cut-off

| **Delay** | **Cancer** | | **p-value°** |
| --- | --- | --- | --- |
|  | **Yes (n=18)** | **No (n=49)** |  |
| ***Diagnostic**** | | | |
| 1 year: |  |  | 0.706 |
| - ≤1 year | 6 | 14 |  |
| - >1 year | 12 | 35 |  |
| 3 years: |  |  | 0.993 |
| - ≤3 years | 7 | 19 |  |
| - >3 years | 11 | 30 |  |
| 5 years: |  |  | 0.463 |
| - ≤5 years | 7 | 24 |  |
| - >5 years | 11 | 25 |  |
| 10 years: |  |  | 0.142 |
| - ≤10 years | 9 | 34 |  |
| - >10 years | 9 | 15 |  |
| ***Therapeutic**** | | | |
| 1 year: |  |  | 0.756 |
| - ≤1 year | 5 | 12 |  |
| - >1 year | 12 | 35 |  |
| 3 years: |  |  | 0.826 |
| - ≤3 years | 6 | 18 |  |
| - >3 years | 11 | 29 |  |
| 5 years: |  |  | 0.502 |
| - ≤5 years | 6 | 21 |  |
| - >5 years | 11 | 26 |  |
| 10 years: |  |  | 0.228 |
| - ≤10 years | 8 | 30 |  |
| - >10 years | 9 | 17 |  |

*Times were calculated from the detected or estimated disease onset data
